# Characterizing the Short-Term Habituation of Event-Related Evoked Potentials

**DOI:** 10.1523/ENEURO.0014-18.2018

**Published:** 2018-09-28

**Authors:** Flavia Mancini, Alessia Pepe, Alberto Bernacchia, Giulia Di Stefano, André Mouraux, Gian Domenico Iannetti

**Affiliations:** 1 Department of Neuroscience, Physiology and Pharmacology, University College London, London WC1E 6BT, United Kingdom; 2 Computational and Biological Learning, Department of Engineering, University of Cambridge, Cambridge CB2 1PZ, United Kingdom; 3 Department of Human Neuroscience, University of Rome “La Sapienza,” Rome 00185, Italy; 4 Institute of Neuroscience, Université Catholique de Louvain, Brussels B-1200, Belgium; 5 Neuroscience and Behaviour Laboratory, Istituto Italiano di Tecnologia (IIT), Rome, Italy

**Keywords:** EEG, ERP, habituation, nociception, somatosensory

## Abstract

Fast-rising sensory events evoke a series of functionally heterogeneous event-related potentials (ERPs). Stimulus repetition at 1 Hz induces a strong habituation of the largest ERP responses, the vertex waves (VWs). VWs are elicited by stimuli regardless of their modality, provided that they are salient and behaviorally relevant. In contrast, the effect of stimulus repetition on the earlier sensory components of ERPs has been less explored, and the few existing results are inconsistent. To characterize how the different ERP waves habituate over time, we recorded the responses elicited by 60 identical somatosensory stimuli (activating either non-nociceptive Aβ or nociceptive Aδ afferents), delivered at 1 Hz to healthy human participants. We show that the well-described spatiotemporal sequence of lateralized and vertex ERP components elicited by the first stimulus of the series is largely preserved in the smaller-amplitude, habituated response elicited by the last stimuli of the series. We also found that the earlier lateralized sensory wave habituates across the 60 trials following the same decay function of the VWs: this decay function is characterized by a large drop at the first stimulus repetition followed by smaller decreases at subsequent repetitions. Interestingly, the same decay functions described the habituation of ERPs elicited by repeated non-nociceptive and nociceptive stimuli. This study provides a neurophysiological characterization of the effect of prolonged and repeated stimulation on the main components of somatosensory ERPs. It also demonstrates that both lateralized waves and VWs are obligatory components of ERPs elicited by non-nociceptive and nociceptive stimuli.

## Significance Statement

Our results provide a functional characterization of the decay of the different event-related potential (ERP) components when identical fast-rising somatosensory (nociceptive and non-nociceptive) stimuli are repeated at 1 Hz. These stimuli elicit ERPs obligatory contributed by both early lateralized components and late vertex components, even when stimulus repetition minimizes stimulus relevance. This challenges the view that lateralized waves are not obligatorily elicited by nociceptive stimuli. Furthermore, the lateralized and vertex waves (VWs) habituate to stimulus repetition following similar decay functions, which are unlikely explained in terms of fatigue or adaptation of skin receptors.

## Introduction

Sudden sensory events evoke a series of transient responses in the ongoing electrocortical activity [event-related potentials (ERPs)]. ERPs are functionally heterogeneous and reflect the activity of distinct cortical generators overlapping in time and space (Sutton et al., 1965). Since these generators include both sensory and associative cortical areas, the scalp distribution of the early lateralized ERP components elicited by stimuli of different modalities partly differs depending on the modality of the sensory input. In contrast, the scalp distribution of the late and largest ERP components is virtually identical regardless of the modality of the eliciting stimulus (Mouraux and Iannetti, 2009): it consists in a biphasic negative-positive deflection widespread over the scalp and maximal at the vertex, often referred to as vertex wave (VW) or vertex potential (Bancaud et al., 1953).

The VW amplitude is maximal when fast-rising stimuli are presented using large and variable inter-stimulus intervals of several seconds (Mouraux and Iannetti, 2009; Huang et al., 2013), or when the stimulus reflects behaviorally relevant changes within a regular series of otherwise identical stimuli (Snyder and Hillyard, 1976; Valentini et al., 2011; Ronga et al., 2013). In contrast, when identical stimuli are monotonously repeated at short and regular intervals (e.g., 0.5 or 1 Hz), the VW amplitude strongly decays (Jasper and Sharpless, 1956; Ritter et al., 1968; Davis et al., 1972; Mouraux and Iannetti, 2009; Liang et al., 2010; Wang et al., 2010). Although the decay of the VW due to repeated stimulation at different frequencies has been described (Fruhstorfer et al., 1970; Greffrath et al., 2007), a formal characterization of how the different constituent components of the ERP habituate over time is still missing. This is particularly important considering that previous studies suggested that neural activity in different cortical regions adapts to repeated stimulation at different timescales: for instance, neural activity in associative regions elicited by trains of innocuous, somatosensory stimuli decays faster than neural activity in sensory cortices (Forss et al., 2001; Venkatesan et al., 2014). However, these results may not generalize to responses elicited by noxious somatosensory stimuli: a previous study has suggested that the repetition of intraepidermal nociceptive stimuli at 1 Hz for 1 min fully suppresses lateralized evoked responses (Mouraux et al., 2013).

Therefore, our primary objective was to describe the short-term habituation of the different constituents of somatosensory nociceptive and non-nociceptive ERPs: both the large centrally-distributed VWs (N2 and P2 waves) and the smaller lateralized somatosensory waves (N1 and P4 waves). These are all the known waves elicited by nociceptive stimulation (Treede et al., 1988; Valentini et al., 2012; Hu et al., 2014). As in Mouraux et al. (2013), we recorded EEG while delivering trains of 60 identical stimuli at 1 Hz. In one group of healthy participants, we transcutaneously and electrically stimulated nerve trunks, activating directly all large-diameter Aβ somatosensory afferents and eliciting non-painful sensations. In a separate group of participants, we used radiant-heat stimuli that selectively activate skin nociceptors and elicit sensations of Aδ-mediated pinprick pain. We did not use intraepidermal electrical stimulation of nociceptive afferents (Mouraux et al., 2013), because it can induce strong habituation of peripheral nociceptors (the stimulus is delivered always in the same location, whereas radiant heat stimuli can be easily displaced to reduce nociceptor fatigue). The use of two different somatosensory stimuli allowed to cross-validate and generalize our findings across different sensory pathways.

**Figure 1.**
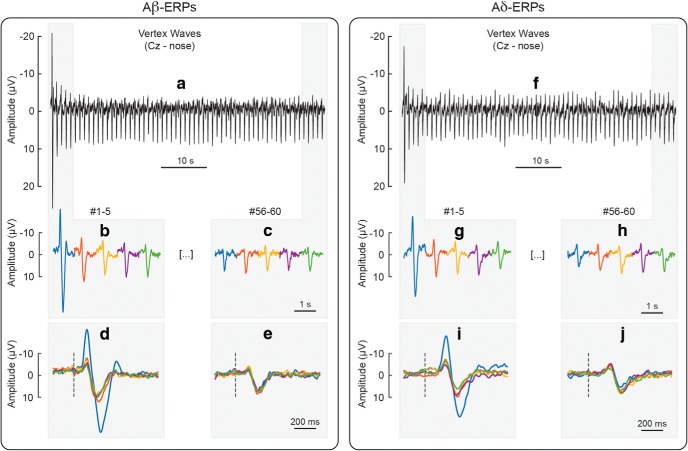
Habituation of VWs (N2, P2) elicited by repeated Aβ (panels ***a–e***) and Aδ stimuli (panels ***f–j***), at electrode Cz referenced to the nose. Panel ***a*** shows the VWs elicited by 60 Aβ stimuli delivered at 1 Hz, whereas panel ***f*** shows the VWs elicited by 60 Aδ stimuli delivered at the same frequency. To facilitate visual comparison, the figure displays, enlarged and concatenated, the responses to the first five Aβ stimuli (panel ***b***), the last five Aβ stimuli (panel ***c***), the first five Aδ stimuli (panel ***g***), and the last five Aδ stimuli (panel ***h***). The figure also displays, enlarged and superimposed, the same responses to the first five Aβ stimuli (panel ***d***), the last five Aβ stimuli (panel ***e***), the first five Aδ stimuli (panel ***i***), and the last five Aδ stimuli (panel ***j***).

**Figure 2.**
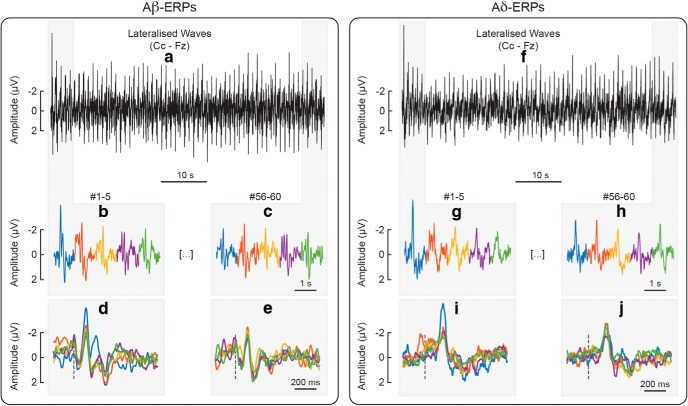
Habituation of the lateralized somatosensory waves (N1, P4) elicited by repeated Aβ (panels ***a–e***) and Aδ stimuli (panels ***f–j***), at the central electrode contralateral to hand stimulation (Cc) referenced to Fz. Panel ***a*** shows the lateralized waves elicited by 60 Aβ stimuli delivered at 1 Hz, whereas panel ***f*** shows the lateralized waves elicited by 60 Aδ stimuli delivered at the same frequency. To facilitate visual comparison, the figure displays, as enlarged and concatenated, the responses to the first five Aβ stimuli (panel ***b***), the last five Aβ stimuli (panel ***c***), the first five Aδ stimuli (panel ***g***), and the last five Aδ stimuli (panel ***h***). The figure also displays, as enlarged and superimposed, the same responses to the first five Aβ stimuli (panel ***d***), the last five Aβ stimuli (panel ***e***), the first five Aδ stimuli (panel ***i***), and the last five Aδ stimuli (panel ***j***).

**Figure 3.**
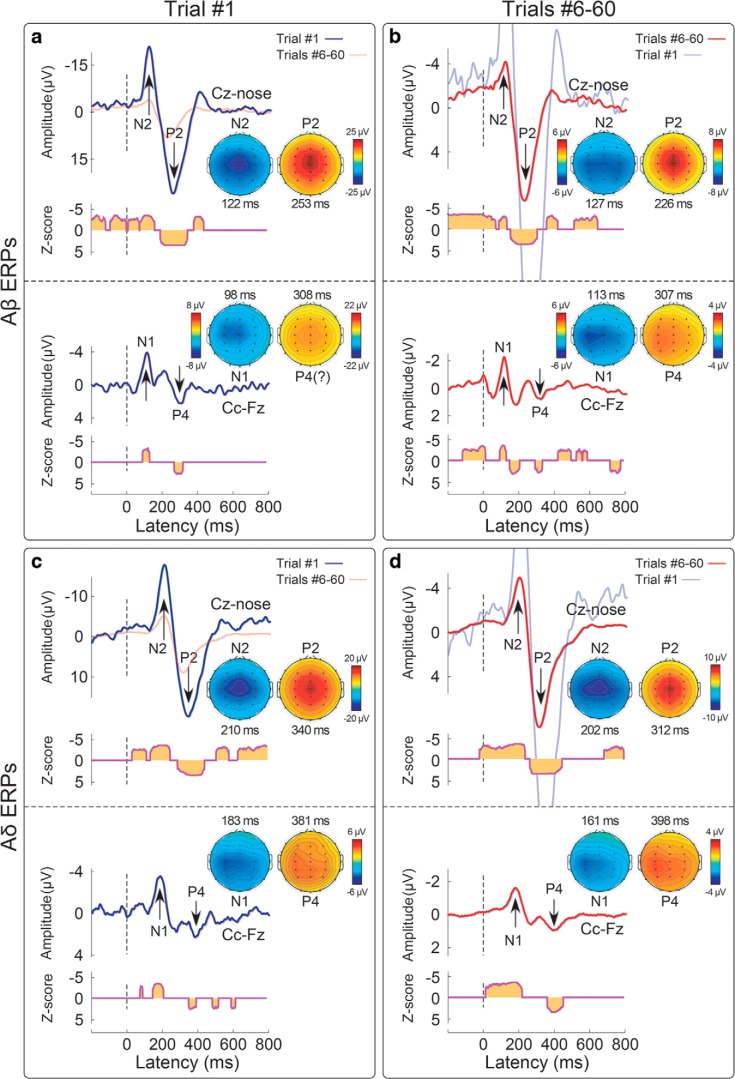
Habituation of VWs (N2, P2) and lateralized responses (N1, P4) elicited by Aβ (panels ***a***, ***b***) and Aδ (panels ***c***, ***d***) somatosensory stimuli. Displayed signals show group-level ERPs recorded from the vertex (Cz vs nose) and from the central electrode contralateral to the stimulated hand (Cc vs Fz), elicited by the first stimulus in a series (non-habituated response; panels ***a***, ***c***) and by the average of trials #6–#60 (habituated response; panels ***b***, ***d***). Scalp topographies (signals referenced to the nose) are displayed at the peak latency of the N1, N2, P2, and P4 waves, in all conditions. The N1, N2, and P2 waves were significantly >0 both in trial #1 and in trials #6–#60, as shown by the point-by-point, cluster-corrected (*p* < 0.05, two tails), one-sample Wilcoxon z-statistics plotted below each ERP wave.

**Figure 4.**
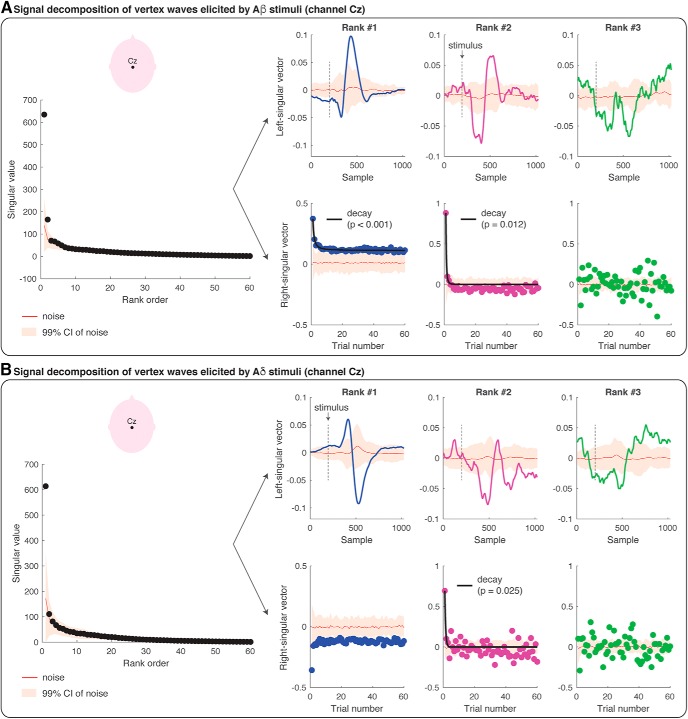
SVD and modeling of the amplitude modulation of the VWs (at channel Cz) elicited by repeated Aβ (panel ***A***) and Aδ (panel ***B***) stimuli. Each figure panel displays the singular values at each of the 60 ranks, and the left- and right-singular vectors at the first three ranks. The singular values are the scaling factors of left- and right-singular vectors, and they are ranked according to their importance (from the most important to the least important). The left-singular vector shows the modulation of EEG amplitude across the epoch of 1000 ms (i.e., 1024 samples recorded at 1024 Hz). The stimulus onset is marked with a dashed black line. The right-singular vector shows the modulation of EEG amplitude across the 60 trials. The red line in all plots shows the group-average results of the SVD of the single-subject residual noise traces, with a 99% confidence interval for statistical comparison (*p* = 0.01). Habituation models were fitted to the right-singular vectors at each rank. If a habituation model wins over a non-habituation model, the fit of the model is displayed with a black line superimposed on the right-singular vector values and the corresponding *p* value is reported. In all the instances in which the non-habituation model wins over a habituation model, no fit is displayed.

We addressed two complementary questions. First, we statistically assessed whether the main response components were present in both the non-habituated ERP (i.e., the ERP elicited by the first stimulus of a series) and the habituated ERP (i.e., the ERP elicited by later stimuli that yield a stable, habituated response). The rationale for this decision was the consistent observation that the amplitude of the main ERP waves (i.e., VWs) decays only minimally after the first few stimulus repetitions (Ritter et al., 1968; Fruhstorfer et al., 1969, 1970; Fruhstorfer 1971; Greffrath et al., 2007; Mouraux et al., 2013), a finding corroborated by the present results (Figs. 1–4). Second, we asked whether and how the lateralized and VWs habituated throughout the block of 60 stimuli. We used singular value decomposition (SVD) to separate the ERP waveform from its amplitude change across stimulus repetitions. SVD provides a small number of components that best approximate the data and explain most of its variance (Golub and Reinsch, 1970). This approach allowed us to investigate the decay function of small ERP components, such as the lateralized waves.

## Materials and Methods

### Participants

Thirty-two healthy subjects (14 women) aged 19–31 years (mean ± SD: 23.6 ± 3.9) participated in the study, after having given written informed consent. All experimental procedures were approved by the ethics committee of University College London (2492/001).

### Transcutaneous electrical stimulation of Aβ fibers

Innocuous stimulation of Aβ afferents consisted of square-wave pulses (100-µs duration), generated by a constant current stimulator (DS7A, Digitimer). Stimuli were delivered through a bipolar electrode placed above the superficial radial nerve and elicited a paresthetic sensation in the corresponding innervation territory. Aβ detection thresholds were identified using the method of ascending staircases, on the right hand. The detection threshold was defined as the average of the lowest stimulus energy eliciting a sensation in three consecutive trials. Electrical stimuli were delivered at ∼300% of each individual’s Aβ detection threshold. Stimulus intensity was slightly adjusted to elicit sensations of comparable intensities on the left and right hands (mean ± SD, 17.4 ± 11.4 mA) and to make sure that the elicited sensation was never painful.

### Cutaneous laser stimulation of Aδ and C fibers

Nociceptive stimuli were radiant heat pulses generated by an infrared neodymium:yttrium-aluminum-perovskite laser with a wavelength of 1.34 µm (Nd:YAP; Electronical Engineering). At this wavelength, laser pulses excite Aδ and C nociceptive free nerve endings in the epidermis directly and selectively, i.e., without coactivating touch-related Aβ fibers in the dermis (Bromm and Treede, 1984; Baumgärtner et al., 2005; Mancini et al., 2014). The duration of each laser pulse was 4 ms.

Laser stimuli were delivered within a squared skin area (4 × 4 cm) centered on the dorsum of the hand, encompassing the area in which the stimulation of Aβ afferents elicited the paresthesia. The laser beam was transmitted through an optic fiber, and its diameter at target site was set at ∼6 mm by focusing lenses. A visible He–Ne laser pointed to the stimulated area, within which the laser beam was manually displaced after each stimulus. The laser was triggered by a computer script.

The method of ascending staircases used for identifying the detection threshold of Aβ stimuli was also used to identify the detection threshold of Aδ stimuli. For the EEG recordings, the stimulus energy was clearly above the activation threshold of Aδ fibers (0.53 ± 0.06 J/mm^2^). This stimulus energy elicited intense but tolerable pinprick pain sensations, of comparable intensities on the right and left hands. Because variations in baseline skin temperature may modulate the intensity of the afferent nociceptive input (Iannetti et al., 2004), an infrared thermometer was used to ensure that the hand temperature varied no >1°C across blocks. To avoid receptor fatigue or sensitization, the laser beam was displaced after each stimulus by ∼1 cm within the predefined stimulated area.

### Experimental procedure

Participants sat comfortably with their hands resting on a table in front of them. They were instructed to focus their attention on the stimuli and fixate a yellow circular target (diameter: 1 cm) placed in front of them at a distance of ∼60 cm from their face. A black curtain blocked the view of the hands. Throughout the experiment, white noise was played through headphones, to mask any sound associated with the either type of somatosensory stimulation.

The experiment was performed on 32 participants, divided in two groups of 16 participants. One group received electrical stimuli, and the other group received laser stimuli, using an identical procedure. Each participant received the somatosensory stimuli in 10 blocks, separated by a 5-min interval, during which participants were allowed to rest. Each block consisted of 60 somatosensory stimuli delivered at 1 Hz: thus, each block lasted 1 min. In each block, stimuli were delivered either to the right hand or to the left hand. Right- and left-hand blocks were alternated. The order of blocks was balanced across participants; half of the subjects started with a right-hand block, and the other half started with a left-hand block. At the end of each block, participants were asked to provide an average rating of perceived stimulus intensity, with reference to the modality of the stimulus and using a numerical scale ranging from 0 (“no shock sensation” or “no pinprick sensation”) to 10 (“most intense shock sensation” or “most intense pinprick sensation”). This was done to ensure that the perceived intensity of the stimuli was similar across blocks (rating variability, SD across blocks: electrical stimuli, 0.2 ± 0.2; laser stimuli: 0.3 ± 0.4).

### Electrophysiological recordings

EEG was recorded using 30 Ag–AgCl electrodes placed on the scalp according to the International 10-20 system (Electro-Cap International), using the nose as reference. Electrode positions were Fp1, Fpz, Fp2, F7, F3, Fz, F4, F8, T3, C3, Cz, C4, T4, T5, P3, Pz, P4, T6, O1, Oz, O2, FCz, FC4, FC3, Cp3, Cp4. Eye movements and blinks were recorded from the right orbicularis oculi muscle, using two surface electrodes. The active electrode was placed below the lower eyelid, and the reference electrode a few centimeters laterally to the outer canthus. Signals were amplified and digitized using a sampling rate of 1024 Hz (SD32; Micromed).

### EEG analysis

#### Preprocessing

EEG data were preprocessed and analyzed using MATLAB R2016b, Letswave 6 and EEGLAB (https://sccn.ucsd.edu/eeglab/). Continuous EEG data were bandpass filtered from 0.5 to 30 Hz using a Butterworth filter, segmented into epochs using a time window ranging from -0.2 to 0.8 s relative to the onset of each stimulus, and baseline corrected using the interval from -0.2 to 0 s as reference. Trials contaminated by large artefacts (<10% per condition) were removed. Eye blinks and movements were corrected using a validated method based on unconstrained independent component analysis (“runica” algorithm of EEGLAB). In all datasets, independent components related to eye movements showed a large EOG channel contribution and a frontal scalp distribution. To allow averaging across blocks while preserving the possibility of detecting lateralized EEG activity, scalp electrodes were flipped along the medio-lateral axis for all signals recorded in response to left hand stimulation. Hereinafter, we refer to the central electrode contralateral to the stimulated hand as Cc. In each participant, we averaged each of the 60 ERP responses across the 10 recording blocks, and thus obtained 60 average ERP waveforms: one for each of the 60 trials and for each participant.

#### Statistical assessment of ERP components

We assessed the consistency of stimulus-evoked modulations of EEG amplitude across time, to statistically evaluate whether EEG deflections in the post-stimulus time window (from 0 to +0.8 s) were significantly greater than baseline. Specifically, we performed a one-sample, non-parametric, Wilcoxon signed-rank test against zero for each time point of the entire baseline-corrected, single-subject waveforms, using cluster-level permutation testing (Maris and Oostenveld, 2007; Van Den Broeke et al., 2015). A non-parametric test was chosen over a parametric test to not make the assumption that the data were normally distributed. The nonparametric cluster-based permutation approach for statistical testing assumes that true neural activity will tend to generate signal changes over contiguous time points. First, the EEG waveforms of the different conditions were compared by means of a point-by-point, one-sample Wilcoxon signed-rank test against zero. Then, clusters of contiguous time points above the critical *z* score for a non-parametric two-sided test were identified, and an estimate of the magnitude of each cluster was obtained by computing the sum of the *z* scores constituting each cluster (cluster-level statistic). Random permutation testing (1000 times) of the subject-specific ERP waveforms of the different conditions (performed independently for every subject) was then used to obtain a reference distribution of mean cluster magnitude. Finally, the proportion of random partitions that resulted in a larger cluster-level statistic than the observed one was calculated. Clusters in the observed data were regarded as significant if they had a magnitude exceeding the threshold of the 2.5th and 97.5th percentile (corresponding to a two-sided test). This analysis was performed separately on the non-habituated ERP and on the habituated ERP of each modality.

The non-habituated ERP was derived, for each participant, by averaging the responses elicited by the 1st stimulus of all blocks. The habituated ERP was derived, for each participant, by averaging the responses elicited by the 6th to the 60th stimuli of all blocks. The decision of using these responses elicited by stimuli 6th to 60th as a proxy of the habituated ERP was based on the observation that the amplitude of the main ERP waves decays only minimally after the first five stimulus repetitions, as observed here (Figs. 1, 2, 4) and previously described (Fruhstorfer et al., 1970; Greffrath et al., 2007). Figures 1, 2 show how the amplitude of the ERPs was consistently habituated after the first few stimulus repetitions.

#### Modeling the within-block decay of the lateralized and VWs

We tested whether and how the amplitude of the VWs and of the lateralized wave evoked by Aβ and Aδ stimuli was modulated as a function of stimulus repetition. In each participant, we first averaged each of the 60 ERP responses across the 10 recording blocks, and thus obtained 60 average ERP waveforms: one for each of the 60 trials. Then, we averaged across participants and, for each modality, we obtained 60 group-level averages. To study the amplitude modulation of the entire wave form across 60 trials, we decomposed the EEG signals at electrodes of interest (Cz and Cc) using SVD (Golub and Reinsch, 1970). We used SVD to decompose the modulation of the EEG amplitude across the 1000-ms epoch (which give rise to the ERP wave) from the modulation of the EEG amplitude across 60 trials.

SVD is a method for decomposing the data matrix **M**(*s×e*), in this case EEG signals: *s* = 1024 time samples, *e* = 60 trials (given that the sampling rate is 1024 Hz, each 1000-ms epoch has 1024 samples) into *s* wave components [left singular vectors, defined as the columns of a matrix **U**(*s x s*)] and *e* habituation components [right singular vectors, defined as the columns of a matrix **V**(*e x e*)]. The left-singular vectors tell us how the EEG amplitude is modulated across the 1000-ms epoch (wave component), and the right-singular vectors describe how the EEG amplitude is modulated across 60 trials (habituation component). Each left-right component pair is multiplied by a scaling factor σ, and pairs are rank-ordered according to those factors, where the most important pairs correspond to the largest values of σ, and the least important ones (typically noise) correspond to the lowest σ. Formally, SVD is given by **M** = **U∑V^T^**, where ∑ is a *s x e* diagonal matrix with the scaling factors on the diagonal (singular values), **U** and **V** are the matrices of left and right singular vectors, respectively, and **V^T^** is the matrix transpose of **V**. The first component pair gives the optimal rank-1 approximation to the original data matrix, in the least square sense. The first two components give the optimal rank-2 approximation, and so on and so forth.

To test the significance of the SVD decomposition, we separated the variance caused by stimulus-evoked activity from other types of variance (noise), and performed the SVD on the noise traces; finally, we tested whether the results of the SVD performed on the noise traces were different from the SVD performed on **M** (which contains a mixture of signal and noise), adapting an approach previously described (Sengupta and Mitra, 1999; Machens et al., 2010).

Specifically, for each subject and condition, we first estimated the residual noise traces η_i_ (*s, e*), by taking the average of the differences between the single-subject EEG amplitude y*_i_* (*s, e*) and group-average EEG amplitude Y_-i_ (*s, e*) (the group average was calculated after excluding subject *i*):

η_i_ (*s, e*) = y_i_ (*s, e*) – Y_-i_(*s, e*)

We then performed SVD on the residual noise traces η (*s, e*), for each subject and condition. We averaged the resulting **U_noise_**, **∑_noise_**, **V^T^_noise_** across subjects and divided them by the square root of the number of subjects. We also calculated their SEM. We tested the significance of the ranks of **∑** by comparing whether each diagonal value of **∑** was greater than the corresponding value of [**∑_noise_ +** 2.33 SE]: this corresponds to a one-tail test at a *p* = 0.01. Lastly, we tested the significance of **U** and **V^T^** by comparing whether their value at each rank was different (either greater or lower) than the corresponding value of [**U_noise_** ± 2.58 SEM] and [**V^T^_noise_** ± 2.58 SEM]: this corresponds to a two-tails test at a *p* = 0.01.

Finally, we modeled the amplitude modulation across trials (habituation components) by fitting the following models to the right-singular vectors at each eigenvalue scale factor (or rank order):

(1) y = a + b/x
(2) y = a + b/x^c^
(3) y = a + b *e* ^-cx^
(4) y = c

where *y* is the peak amplitude of each given ERP wave, *x* is the trial number (from 1 to 60), *e* is the Euler constant, and *a, b, c* are the parameters to be estimated using a non-linear least squares method. We tested these specific models of ERP decay (#1–#3) given the previous evidence that the VW decays sharply at the first stimulus repetition (Fruhstorfer et al., 1970; Greffrath et al., 2007; Iannetti et al., 2008; Valentini et al., 2011; Ronga et al., 2013). Note that model (4) corresponds to the absence of habituation, and fitting this model simply gives c equal to the mean of y. To compare which model best fitted the data, we calculated the Bayesian Information Criterion (BIC) of each model for each component, ordered by rank. The BIC allows a fair comparison between models of different complexity because it penalizes models with more parameters (Cover and Thomas, 2006). The lower the BIC, the better the model represents the measured data. For each component rank, we calculated the probability of rejecting the null hypothesis that there was no habituation (i.e., model #4 best represents the data) and accepting the alternative hypothesis that there was significant habituation (i.e., either model #1, #2, or #3 wins), by using a resampling approach with 1000 iterations: at each iteration, we shuffled the order of epochs, fitted models #1–#4, and compared the goodness of fit according to BIC.

#### Code accessibility

The code described above in Statistical assessment of ERP components and Modeling the within-block decay of the lateralized and VWs was written in MATLAB 2016b and is freely available online at https://github.com/flamancini/ERP_habituation_2018. The code is also available as Extended Data.

10.1523/ENEURO.0014-18.2018.ed1 Extended data MATLAB R2016b code for the Statistical assessment of ERP components and Modeling the within-block decay of the lateralized and VWs. Download Extended Data 1, ZIP file.

#### Data availability

Pre-processed EEG data are publicly available at https://osf.io/8wj3s/.

## Results

### Response waveforms and topographies

Group-average ERPs elicited by Aβ and Aδ stimuli are shown in Figures 1–3. As expected, the latency of Aδ-ERPs was longer than the latency of Aβ-ERPs, because Aδ fibers are thinly myelinated and thus have slower conduction velocity than large-myelinated Aβ fibers (Mountcastle, 2005).

Figure 1 shows that the amplitude decay of the negative and positive VWs (N2 and P2, Cz vs nose) elicited by the 60 repeated somatosensory stimuli, whereas Figure 2 shows the amplitude modulation of the lateralized somatosensory waves (N1 and P4, Cc vs Fz). To facilitate visual inspection, we enlarged the responses to the first five and last five stimuli (same responses presented both concatenated and superimposed in Figs. 1, 2). Figure 3 demonstrates that, both in the non-habituated response (trial #1, Fig. 3*a*,*c*) and in the habituated response (average of trials #6–#60, Fig. 3*b*,*d*), the N2 and P2 waves were greater than baseline. Not only they survived 1-min of repeated stimulation, but clearly dominated the majority of the ERP responses.

In both stimulus modalities, the lateralized somatosensory waves were much smaller than the VWs, as expected (Valentini et al., 2012; Hu et al., 2014), and the identification of the P4 peak was ambiguous for the Aβ-ERP elicited by trials 6–60 (Fig. 3*A*). Importantly, albeit small in amplitude, both the early N1 and the late P4 lateralized waves elicited in trials 1 and 6–60 were nevertheless consistently greater than baseline, as demonstrated by the point-by-point non-parametric tests reported in Figure 3. The peaks of the N1 waves elicited in trials 1 (Fig. 3*a*,*c*) and 6–60 (Fig. 3*b*,*d*) had maximal spatial distribution over the central electrodes in the hemisphere contralateral to hand stimulation (Fig. 3), as shown in previous studies (Hu et al., 2014; Mancini et al., 2015).

### Modeling the within-block decay of the lateralized waves and VWs

**Figure 5.**
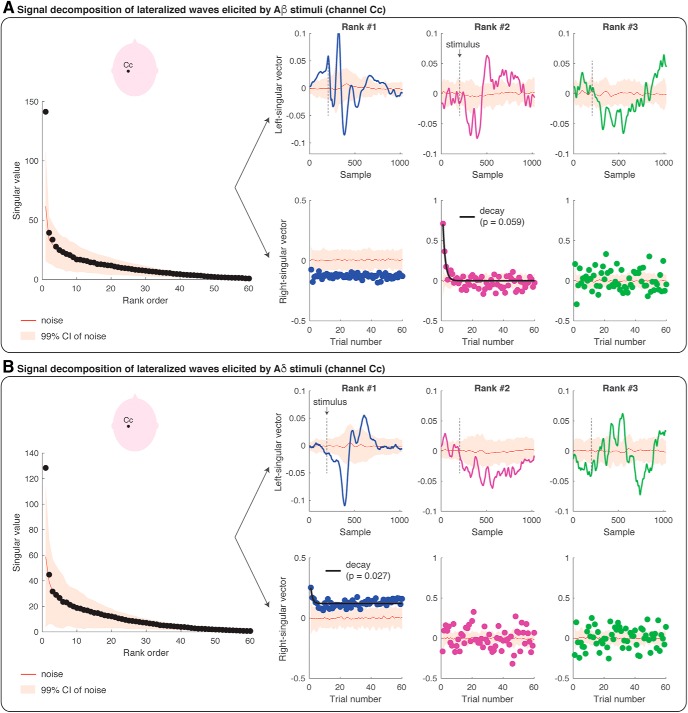
SVD and modeling of the amplitude modulation of the lateralized waves (at channel Cc) elicited by repeated Aβ (panel ***A***) and Aδ (panel ***B***) stimuli. Each figure panel displays the singular values at each of the 60 ranks, and the left- and right-singular vectors at the first three ranks. The singular values are the scaling factors of left- and right-singular vectors, and they are ranked according to their importance (from the most important to the least important). The left-singular vector shows the modulation of EEG amplitude across the epoch of 1000 ms (i.e., 1024 samples recorded at 1024 Hz). The stimulus onset is marked with a dashed black line. The right-singular vector shows the modulation of EEG amplitude across the 60 trials. The red line in all plots shows the group-average results of the SVD of the single-subject residual noise traces, with a 99% confidence interval for statistical comparison (*p* = 0.01). Habituation models were fitted to the right-singular vectors at each rank. If a habituation model wins over a non-habituation model, the fit of the model is displayed with a black line superimposed on the right-singular vector values and the corresponding *p* value is reported. In all the instances in which the non-habituation model wins over a habituation model, no fit is displayed.

**Figure 6.**
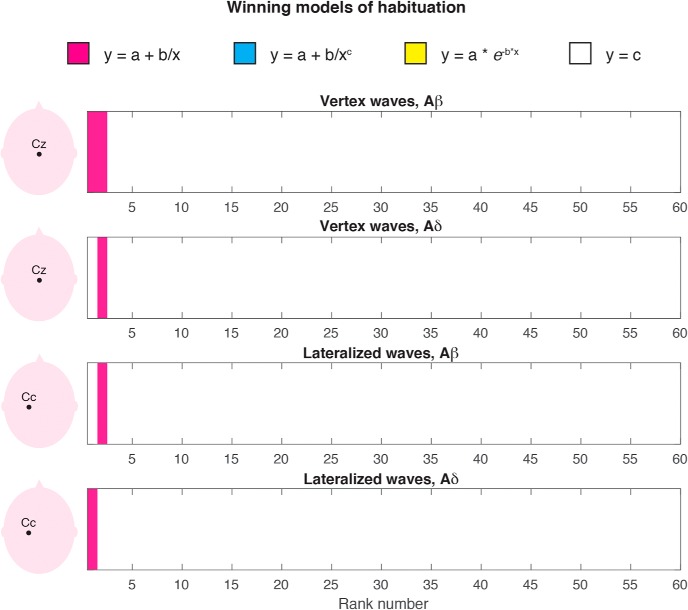
Winning model of ERP modulation by stimulus repetition. Following SVD, three habituation models and a non-habituation model were fitted to the right-singular vectors at each of the 60 ranks and compared according to BIC. The winning models are color-coded (pink: *y = a +b/x*; white: no habituation). Other decay models never win (blue, yellow).

We took a modeling approach to decompose the modulation of the EEG amplitude across the 1000-ms epoch (which give rise to the ERP wave) from the modulation of the EEG amplitude across 60 trials. This analysis has the benefit of providing an optimal, rank-based approximation to the original data matrix, allowing us to detect habituation effects. Figures 4, 5 display the results of the SVD analyses performed at channels Cz (VWs) and Cc (lateralized waves) respectively, elicited by non-nociceptive Aβ stimuli (Figs. 4*a*, 5*a*) and nociceptive Aδ stimuli (Figs. 4*b*, 5*b*). The singular values can be considered as the scaling factors of the left-singular and right-singular vectors. The left-singular vector shows whether and how the EEG amplitude was modulated within the 1000-ms epoch, and right-singular vector shows whether and how the EEG amplitude was modulated across 60 trials. The noise distribution for singular, left-singular, and right-singular vectors is shown in red (with 99% confidence intervals). Figure 6 summarizes which model best fitted the EEG amplitude modulation across trials, at each rank and according to BIC.

The amplitude modulation of the VWs elicited by Aβ stimuli was significantly described by the first two ranks (Fig. 4*A*): the first two singular values were greater than the singular values of the noise distribution (at *p* = 0.01). The modulation of the EEG amplitude within the epoch (left-singular vectors) had the characteristic shape of the VW at the first two ranks (Fig. 4*A*). The latency of the peaks of these waveforms fell clearly within the range of the N2 and P2 peak latencies (Fig. 4*A*, left-singular vector; compare Fig. 3*a*,*b*): the peaks of the left-singular vector at the first rank had a latency of 125 ms (corresponding to the Aβ-N2 peak) and 225 ms (corresponding to the Aβ-P2 peak); the peaks at the second rank had a latency of 196 ms (corresponding to the late part of the Aβ-N2 wave) and 292 ms (corresponding to the late part of the Aβ-P2 wave). Furthermore, the EEG amplitude elicited by Aβ stimuli decayed significantly across trials at the first two ranks (Fig. 4*A*, right-singular vector). The winning decay models (*y = a + b/x*) are displayed with a black line superimposed onto the right-singular vectors, and their *p* values were <0.001 at rank-1 and 0.012 at rank-2.

The signal decomposition of the VWs elicited by nociceptive Aδ stimuli is reported in Figure 4*B*. Only the first rank of singular values was greater than noise: at the first rank, the modulation of the EEG amplitude had the characteristic shape and latency of the VW (Fig. 4*B*, left-singular vector): the peaks of the left-singular vector at the first rank had a latency of 202 ms (corresponding to the peak of the Aδ-N2) and 317 ms (corresponding to the peak of the Aδ-P2; compare Fig. 3*c*,*d*). Although the EEG amplitude clearly decreased from the first to the second trial at the first rank, the fitting of decay models was not significant (Fig. 4*B*, right-singular vector). Although the second rank of singular values was not significantly different from noise, the modulation of EEG amplitude across time samples was greater than noise at a latency of 270 ms (corresponding to the late part of the N2 wave) and 380 ms (Fig. 4*B*, left-singular vector): the amplitude of the second-rank component was greater than noise only at the first trial, and its decay was best modeled by the same decay function that described the decay of the VW elicited by Aβ stimuli (*y = a + b/x; p* = 0.025).

The amplitude modulation of the lateralized somatosensory waves elicited by Aβ stimuli (Fig. 5*A*) and Aδ stimuli (Fig. 5*B*) was described by the first rank of singular values (p < 0.01). At the first rank, the peak of the left-singular vector fell within the range of the peak amplitude of the N1 wave, both for Aβ stimuli (112 ms) and for Aδ stimuli (181 ms; Fig. 5*A*,*B*; compare Fig. 3). At the second-rank, the left-singular vector for Aβ stimuli was characterized by two peaks significantly greater than noise: the earliest peak latency fell within the range of the Aβ-N1 peak latency (112 ms), whereas the second peak had a latency longer than the Aβ-N2 and shorter than the Aβ-P2 peaks (184 ms; compare Fig. 3*a*,*b*). The amplitude of the EEG responses elicited by Aβ stimuli at the first rank was greater than noise (Fig. 5*A*, right-singular vector), but did not habituate across trials (i.e., the non-habituation model best fitted the right-singular vector). However, at the second rank, the EEG amplitude of the first three trials was greater than noise, and the signal habituation was again in the form of *y = a + b/x* (Fig. 5*A*, right-singular vector; *p* = 0.059). Finally, the ERP elicited by Aδ stimuli significantly habituated across trials: indeed, the right-singular vector at the first rank habituated following the same decay functions of the N2 and P2 waves elicited by Aβ stimuli and Aδ stimuli (*y = a + b/x*; *p* = 0.027; see also Fig. 6).

## Discussion

In this study, we characterized the habituation of the different components of the ERPs elicited by 60 identical somatosensory stimuli (activating either Aβ non-nociceptive or Aδ nociceptive primary afferents) delivered at 1 Hz. Although the response amplitude was clearly reduced, the spatiotemporal sequence of the ERP waves was overall preserved in the habituated response (Fig. 3). This was substantiated by point-by-point statistical analysis: both lateralized somatosensory components and supramodal vertex components typically observed in the ERP elicited by sporadic and unpredictable stimuli (Liang et al., 2010; Hu et al., 2014; Mancini et al., 2015) also contributed to the ERP elicited by frequent and predictable stimuli. This result challenges a previous report that 60 repetitions of nociceptive stimuli at 1 Hz fully suppresses lateralized waves (Mouraux et al., 2013) and indicates that lateralized waves are obligatorily elicited by nociceptive-selective stimulation. Furthermore, we used SVD to decompose the modulation of the EEG amplitude across the 1000-ms epoch (which give rise to the ERP wave) from the modulation of the EEG amplitude across 60 trials. We found that the same model described the habituation of the VWs and lateralized waves elicited by Aβ and Aδ stimuli (Figs. 4–6): that was the simplest decay function in the form of *y = a + b/x*, where *y* is the EEG amplitude, *x* is the trial number, and *a, b* are the estimated parameters. This indicates that the amplitude of both vertex and lateralized waves decays monotonically, with a largest, transient drop of response magnitude at the first stimulus repetition, followed by much smaller decreases in subsequent repetitions.

### Effect of stimulus repetition on somatosensory lateralized responses

In somatosensory ERPs, the VW is both preceded and followed by other deflections of smaller amplitude. These have a topographical distribution maximal over centro-parietal electrodes in the hemisphere contralateral to hand stimulation. The earliest negative wave is usually referred to as N1 (Treede et al., 1988; Valentini et al., 2012) and the latest positive wave form of somatosensory ERPs is referred to as P4 (Hu et al., 2014; Mancini et al., 2015). Whereas the P4 has only been recently identified and its significance is not yet understood, the N1 has been described repeatedly in a large body of studies (Treede et al., 1988; Spiegel et al., 1996; Garcia-Larrea et al., 2003; Lee et al., 2009; Hu et al., 2014; Mancini et al., 2015), and largely reflects somatosensory-specific neural activities (Lee et al., 2009; Liang et al., 2010).

The neural origin of the N1 wave has been long debated and remains unresolved, but it seems to be at least partially different in the ERPs elicited by non-nociceptive and nociceptive somatosensory stimuli (Garcia-Larrea et al., 2003; Ohara et al., 2004; Frot et al., 2013). A number of studies performing intracerebral recordings have indicated that the Aδ-N1 wave is largely contributed by the operculo-insular cortex (Frot et al., 1999; Peyron et al., 2002; Valeriani et al., 2004), whereas other studies have indicated that both the N1 and P4 waves can also be generated in the primary somatosensory cortex, both in human EEG and rodent ECoG recordings (Treede et al., 1988; Valentini et al., 2012; Hu et al., 2014; Jin et al., 2018). For instance, a previous EEG study (Valentini et al., 2012) has demonstrated that the N1 elicited by nociceptive stimulation of the right and left hand have maximum scalp distribution over the central-parietal electrodes contralateral to the stimulated side. In contrast, the N1 elicited by nociceptive stimulation of the right and left foot are symmetrically distributed over the central-parietal midline electrodes (see also Treede et al., 1988; Jin et al., 2018). These findings are compatible with the somatotopic representation of the body in the primary somatosensory and motor cortex.

A novel result of our study is that these somatosensory N1 and P4 responses are detectable not only in the response to the first stimulus, but also in the habituated ERP response, as supported by the statistical assessment of the scalp distribution of the ERP response elicited by both the first and the last stimuli of the series (Fig. 3). This is important, given that a previous study using trains of intraepidermal electrical shocks at 1 Hz failed to observe any lateralized response (Mouraux et al., 2013). We note, however, that in this previous study nociceptive afferents were activated using intraepidermal electrical stimulation, which can cause strong peripheral and perceptual habituation, more significant than for radiant heat stimulation (Mouraux et al., 2010). Thus, in Mouraux et al. (2013) peripheral habituation induced by repeated intraepidermal electrical stimulation in the same skin location may have further reduced the already low signal-to-noise ratio of N1 and P4 waves.

Another novel result of our study is that the lateralized waves habituate across the 60 trials following the same decay functions of the VWs (Figs. 4–6). We used SVD not only to decompose the modulation of EEG amplitude within the block and across trials, but also to model the decay of an optimized model of EEG modulation. Indeed, SVD allows separating signals from noise (similarly to principal component analysis) and provides an optimized description of the ERP waves at the most informative ranks. This signal optimization allows characterizing the amplitude modulation of small and noisy ERP components.

A previous MEG study has reported that neural activity originating from primary somatosensory cortex is more resilient to stimulus repetition (2-Hz pneumatic stimulation of the fingers and face): in other words, it decays to a less extent and more slowly than neural activity in higher-order cortical regions, such as the posterior parietal cortex (Venkatesan et al., 2014). We used slower stimulus frequencies than these studies, so we cannot exclude that different time-scales of habituation may emerge at faster stimulus repetitions.

Finally, our design was not suited to investigate the habituation of the earliest sensory components of Aβ-ERPs (e.g. the N20 wave), which typically require averaging responses elicited by hundreds of stimuli. However, we note that the N20 wave of Aβ-ERPs, which originates in area 3b, is very resilient to stimulus repetition (García Larrea et al., 1992) and is not modulated by selective spatial attention (García-Larrea et al., 1991). In contrast, the later N1 waves of Aβ- and Aδ-ERPs can be modulated by spatial attention (Legrain et al., 2002).

### Effect of stimulus repetition on vertex ERP responses

The negative-positive VW is the largest component of the EEG response elicited by sudden sensory stimuli. Converging evidence indicates that stimuli of virtually all sensory modalities can elicit a VW, provided that they are salient enough (Liang et al., 2010). It is therefore not surprising that the VW elicited by auditory stimuli repeated at 1 Hz decays following a function similar to the one observed here for somatosensory stimuli (Fruhstorfer et al., 1970). Even when considering experimental observations that did not formally model the response habituation, the maximum decrease in VW amplitude consistently occurs at the first stimulus repetition, for auditory (Ritter et al., 1968; Fruhstorfer et al., 1970), somatosensory (Larsson, 1956; Fruhstorfer, 1971; Iannetti et al., 2008; Wang et al., 2010; Valentini et al., 2011; Ronga et al., 2013), and visual stimuli (Courchesne et al., 1975; Wastell and Kleinman, 1980). The similarity of the decay of the VW elicited by Aβ and Aδ stimuli (Figs. 1, 3, 4) further supports the multimodal nature of the neural generators of these signals (Mouraux and Iannetti, 2009). The mechanisms underlying such sharp reduction of response amplitude at the first stimulus repetition are likely to be similar across sensory systems.

Before discussing the contribution of the present results in elucidating the functional significance of the VW, it is important to highlight the empirical evidence that the observed response habituation is not due to neural refractoriness of afferent neurons or to fatigue of primary receptors. A previous study recorded ERPs elicited by pairs of nociceptive stimuli delivered at short intervals, which could be either identical or variable across the block (Wang et al., 2010). Only when the inter-stimulus interval was constant across the block, the VWs elicited by the second stimulus were reduced in amplitude. The peak amplitude of the VWs elicited by the second stimulus was instead as large as the VWs elicited by the first stimulus when the inter-stimulus interval was variable, indicating that neither neural refractoriness nor fatigue can easily explain the sharp response decay to stimulus repetition.

Furthermore, if the sharp response habituation at the first stimulus repetition was determined by fatigue of primary sensory receptors, we would have observed different decay profiles for stimuli delivered in varying versus constant spatial locations. Indeed, the VW elicited by contact heat stimuli at long and variable intervals (8–10 s) decays much faster if the second stimulus is delivered at the same spatial location of the first (Greffrath et al., 2007). Instead, we observed remarkably similar patterns of ERP decay for both Aδ laser stimuli delivered at different spatial locations and Aβ electrical stimuli delivered in the same skin region. Additionally, electrical stimuli activate directly the axons in the nerve trunk, bypassing the receptor, further ruling out receptor fatigue as explanation for the Aβ-ERP habituation. Receptor fatigue might still contribute to the slow decrease in ERP magnitude observed across dozens of stimulus repetitions of laser stimuli (Greffrath et al., 2007), but certainly not to the dramatic reduction of ERP amplitude we observed after one single stimulus repetition.

The physiological significance of the VW remains to be properly understood. However, there is evidence that this large electrocortical response reflects neural activities related to the detection of salient environmental events (Jasper and Sharpless, 1956; Mouraux and Iannetti, 2009) and execution of defensive movements (Moayedi et al., 2015; Novembre et al., 2018). The detection of salient events relies on a hierarchical set of rules that consider both their probability of occurrence and their defining basic features (Legrain et al., 2002; Wang et al., 2010; Valentini et al., 2011; Ronga et al., 2013; Moayedi et al., 2016). The present results are informative with respect to this functional framework. Indeed, stimulus repetition did not abolish the VW elicited by either Aβ or Aδ stimuli, although it reduced its amplitude already after the first stimulus repetition. Therefore, even when stimulus saliency is reduced by contextual factors, there is a residual activity of the VW generators, only minimally reduced after the first few stimulus repetitions (Figs. 1, 3*b*,*d*). These findings point toward the existence of an obligatory VW activity triggered by any sudden and detectable change in the environment, even when contextual modulations minimize its behavioral relevance.

Extensive evidence from cell physiology indicates that neural habituation to repeated stimuli arises from alterations of synaptic excitability. Even the simple gill-withdrawal reflex in *Aplysia* dramatically habituates at the first stimulus repetition (Byrne et al., 1978), due to a decreased drive from the sensory neurons onto follower motor neurons (Castellucci et al., 1970; Carew and Kandel, 1973). The temporal profile of this short-term habituation follows a fast decay function (Carew and Kandel, 1973), strikingly similar to that observed in this and other studies on the habituation of electrocortical responses in humans (Fruhstorfer et al., 1970; Greffrath et al., 2007). These synaptic changes have been interpreted as a hallmark of learning, and are central to the ability of the nervous system to adapt to environmental events (Carew and Kandel, 1973). Interpreting the decay of neural responses as functionally relevant for learning is not in contradiction with attentional interpretations: stimuli that are learned and recognized are likely to require less attentional resources than novel stimuli, and stimuli that need to be learned are typically more salient.

### Conclusion

In conclusion, our results provide a functional characterization of the decay of the different ERP components when identical somatosensory stimuli are repeated at 1 Hz. Nociceptive and non-nociceptive stimuli elicit ERPs that are obligatorily contributed by both lateralized and vertex components, even when stimulus repetition minimizes stimulus relevance. This challenges the view that lateralized waves are not obligatorily elicited by nociceptive stimuli. Furthermore, both the lateralized and the VWs habituate to stimulus repetition following similar decay functions, which most possibly cannot be explained in terms of fatigue or adaptation of skin receptors.

## Synthesis

Reviewing Editor: William Stacey, University of Michigan Decisions are customarily a result of the Reviewing Editor and the peer reviewers coming together and discussing their recommendations until a consensus is reached. When revisions are invited, a fact-based synthesis statement explaining their decision and outlining what is needed to prepare a revision will be listed below. The following reviewer(s) agreed to reveal their identity: massimiliano valeriani, Maria Stavrinou.

The paper has made significant improvements and both reviewers were positive about the results. However, both reviewers still had some significant concerns that need to be resolved.

1. This paper posits that the N1 is exclusively generated in S1 area. This is not necessarily true. It is supported by a single paper (Valentini 2012). The paper should acknowledge a wealth of previous information suggesting that the opercular cortex is the main generator (Valeriani et al., 1996, Valeriani et al., 2000, Frot 1999, 2001, 2003, Peyron 2002). Moreover Valeriani et al. (2004) could not record any LEP component from the area 3b by intracortical recording in a patient undergoing stereo-EEG. This conclusion needs to be limited and should be reconciled with the previous work.

2. The most critical concern is about how statistical significance was assessed with the Singular Value Decomposition. The use of the SVD should be justified and introduced in the introduction. This is a major concern because it does not appear to be validated, and without that the data and conclusions cannot be proven. You should take care to describe why you are using it, then make it very clear how the method is validated and how statistical significance was proven.

3. The descriptions of figures need to be improved. Figures 1-2 should also have panels and the text in article should precisely point to Figure X panel I for example. (lines 295-301 is an example of where this is needed). Moreover what the figures contain is not fully stated, for example what T denotes, how it is calculated and what the limits on the y-axis denote?

4. T-tests are used, but first the data should be tested to assure normal distribution.

5. Minor issues:

The modeling equations are not visible in article text.

What is the Cc electrode? It is central contralateral but which one?

Lines 338-345: here the authors should explain what latencies could correspond to which wave. For example latencies 218-270 ms correspond to N2 and p2 (or N2 in the different blocks?).

Lines 519-521: I would use the expression “which most possibly” instead of “which cannot be explained in terms of fatigue or adaptation of skin receptors”.

6. The code is better explained now in the github repository but still after each step the authors could include the function that one should run to run the code and its inputs.

## Author Response

Dear Professor Stacey,

dear Reviewers,

We are grateful for the constructive and thoughtful reviews received. Please find below a point-by-point answer to the “synthesis of reviews”. All changes in the manuscript are highlighted in red and underlined.

With kind regards,

Flavia Mancini

***

Synthesis Statement for Author (Required): The paper has made significant improvements and both reviewers were positive about the results. However, both reviewers still had some significant concerns that need to be resolved. 1. This paper posits that the N1 is exclusively generated in S1 area. This is not necessarily true. It is supported by a single paper (Valentini 2012). The paper should acknowledge a wealth of previous information suggesting that the opercular cortex is the main generator (Valeriani et al., 1996, Valeriani et al., 2000, Frot 1999, 2001, 2003, Peyron 2002). Moreover Valeriani et al. (2004) could not record any LEP component from the area 3b by intracortical recording in a patient undergoing stereo-EEG. This conclusion needs to be limited and should be reconciled with the previous work.

R. We agree that the neural origin of the N1 is very controversial, and we have now made clear that the N1 is not exclusively generated in S1.

We have rephrased the relevant paragraph as follows (lines 437-449):

The neural origin of the N1 wave has been long debated and remains unresolved, but it seems to be at least partially different in the ERPs elicited by non-nociceptive and nociceptive somatosensory stimuli (Garcia-Larrea et al., 2003; Ohara et al., 2004; Frot et al., 2013). A number of studies performing intra-cerebral recordings have indicated that the Aδ-N1 wave is largely contributed by the operculo-insular cortex (Frot et al., 1999; Peyron et al., 2002; Valeriani et al., 2004), whereas other studies have indicated that both the N1 and P4 waves can also be generated in the primary somatosensory cortex, both in human EEG and rodent ECoG recordings (Treede et al., 1988; Valentini et al., 2012; Hu et al., 2014; Jin et al., 2018). For instance, a previous EEG study (Valentini et al., 2012) has demonstrated that the N1 elicited by nociceptive stimulation of the right and left hand have maximum scalp distribution over the central-parietal electrodes contralateral to the stimulated side. In contrast, the N1 elicited by nociceptive stimulation of the right and left foot are symmetrically distributed over the central-parietal midline electrodes (see also Treede et al., 1988; Jin et al., 2018). These findings are compatible with the somatotopic representation of the body in the primary somatosensory and motor cortex.

2. The most critical concern is about how statistical significance was assessed with the Singular Value Decomposition. The use of the SVD should be justified and introduced in the introduction. This is a major concern because it does not appear to be validated, and without that the data and conclusions cannot be proven. You should take care to describe why you

2

are using it, then make it very clear how the method is validated and how statistical

significance was proven.

R. The use of the SVD has now been justified and introduced in the introduction (lines 110-

4). We kindly note that SVD is a well validated and established method for signal

processing, but we agree that we have not proven its statistical significance. We apologise

for this omission, and we have now tested the significance of the SVD with the approach

described at lines 270-290. Results of this analysis are reported at lines 349-401 and in

revised Figures 4-5.

3. The descriptions of figures need to be improved. Figures 1-2 should also have panels and

the text in article should precisely point to Figure X panel I for example. (lines 295-301 is an

example of where this is needed). Moreover what the figures contain is not fully stated, for

example what T denotes, how it is calculated and what the limits on the y-axis denote?

R. We thank the Reviewer for this comment. We have now included panels in Figures 1-2

and improved the figure legends. The methods section describes in detail how the t-tests

yielding the T timecourses were performed.

4. T-tests are used, but first the data should be tested to assure normal distribution.

R. All t-tests were performed for each time point of the single-subject ERP timecourse

(averaged across blocks) and for all electrodes, then corrected for multiple comparison, as

described in the methods. The size of the matrix (32 subjects x 1024 timepoints x 30

electrodes) is too large for meaningful and informative reporting of normality tests at

timepoint-by-timepoint level, but we agree that the inability to report normality tests may

affect their interpretability.

The t-tests that were reported in Figures 1-2 (comparisons between trial 1 vs 2, 2 vs 3, 59 vs

60, 1 vs 60) are now redundant with the more comprehensive SVD and modelling analyses.

The t-test results were initially reported because they offer a more conventional and intuitive

method to preliminarily explore the data. To limit interpretative issues, these t-tests have

now been removed.

The t-tests reported in Figure 3 show comparisons against baseline. In the attempt to

address the reviewer's concern, we have extracted the single-subject peak values of the

components of interest (N1, N2, P2 waves) in each condition and tested whether they

violated normality assumptions using the Shapiro-Wilk test. This procedure was not done for

the P4 wave because its detection can be ambiguous in some subjects, especially in the

habituated response. This is now described at lines 232-243:

“We found moderate-to-strong evidence for normality violation for the Aδ-P2 peak amplitudes at stimuli #1 (p = 0.005)

and #6-60 (p = 0.02). We found no evidence of violation to normality distribution for all other variables (p &gt; 0.05). To

address the two instances of normality violations, we have performed a non-parametric one-sample test (Wilcoxon signed

rank test) on the Aδ-P2 peak values for conditions ”stimulus 1“ and ”stimuli 6-60“, in addition to the point-by-point tstatistics

(Gibbons and Chakraborti, 2011). Both tests provided strong evidence that the Aδ-P2 peak values were greater

than baseline (stimulus 1: z = 3.52, p &lt; 0.001; stimuli 6-60: z = 3.52, p &lt; 0.001), confirming the results of the pointby-

point t-tests reported in Figure 3.”

5. Minor issues:

The modeling equations are not visible in article text.

R. We apologise for this omission, which was simply due to a failure of the pdf conversion. It

has now been rectified.

3

What is the Cc electrode? It is central contralateral but which one?

R. As per 10-20 EEG convention, Cc refers to the central electrode in the hemisphere contralateral to the stimulated hand: therefore, Cc corresponds to C3 for right hand stimulation, and to C4 for left hand stimulation. This has now been clarified throughout the manuscript and in the figure legends.

Lines 338-345: here the authors should explain what latencies could correspond to which wave. For example latencies 218-270 ms correspond to N2 and p2 (or N2 in the different blocks?).

R. We apologise for the lack of precision. This has been now specified.

Lines 519-521: I would use the expression “which most possibly” instead of “which cannot be explained in terms of fatigue or adaptation of skin receptors”.

R. We thank the Reviewer for this suggestion. We have now changed the text accordingly. 6. The code is better explained now in the github repository but still after each step the authors could include the function that one should run to run the code and its inputs.

R. We hope that the documentation is clearer now. Please note that the code can be found as “Extended Data” as part of this submission to eNeuro (it is not yet on github).
